# Association between vitamin A and asthma: A meta-analysis with trial sequential analysis

**DOI:** 10.3389/fphar.2023.1100002

**Published:** 2023-01-30

**Authors:** Jun Hu, Jiajia Sang, Feng Hao, Li Liu

**Affiliations:** ^1^ College of Acupuncture-Moxibustion and Tuina, College of Health Preservation and Rehabilitation, Nanjing University of Chinese Medicine, Nanjing, China; ^2^ Department of Tuina, Affiliated Hospital of Nanjing University of Chinese Medicine/Jiangsu Province Hospital of Chinese Medicine, Nanjing, China; ^3^ Central Laboratory, Affiliated Hospital of Nanjing University of Chinese Medicine/Jiangsu Province Hospital of Chinese Medicine, Nanjing, China

**Keywords:** vitamin A, retinol, asthma, intake, serum concentration

## Abstract

**Objective:** To explore the association between vitamin A (vit A) status and risk of asthma.

**Methods:** PubMed, Web of Science, Embase and the Cochrane Library were electronically searched to identify related studies that reported the association between vit A status and asthma. All databases were searched from inception to November 2022. Two reviewers independently screened literature, extracted data, and assessed risk bias of included studies. Meta-analysis was performed on R software Version 4.1.2 and STATA Version 12.0.

**Results:** A total of 19 observational studies were included. A pooled analysis showed that the serum vit A concentrations in patients with asthma was lower than that in healthy controls (standard mean difference (SMD)= −2.479, 95% confidence interval (CI): −3.719, −.239, 95% prediction interval (PI): −7.510, 2.552), and relatively higher vit A intake in pregnancy was associated with an increased risk of asthma at age 7 years (risk ratio (RR)= 1.181, 95% CI: 1.048, 1.331). No significant correlation was observed between serum vit A levels or vit A intake and the risk of asthma.

**Conclusion:** Our meta-analysis confirms that serum vit A levels are lower in patients with asthma than in healthy controls. Relatively higher vit A intake during pregnancy is associated with an increased risk of asthma at age 7 years. There is no significant correlation between vit A intake and asthma risk in children, nor between serum vit A levels and asthma risk. The effect of vit A may depend on age or developmental stage, diet and genetics. Therefore, further studies are needed to explore the association of vit A and asthma.

**Systematic Review Registration:** https://www.crd.york.ac.uk/prospero/CRD42022358930, identifier CRD42022358930

## Introduction

Asthma is a respiratory disease characterized by recurrent airway obstruction, airway hyperresponsiveness and chronic airway inflammation (Tenero et al., 2018; Alashkar Alhamwe et al., 2020). The prevalence of self-reported, doctor-diagnosed asthma in adults worldwide is 4.3% and varies widely between countries (Papi et al., 2018). Asthma with severe symptoms will lead to respiratory inflammatory stenosis and obstruction, wheezing, dyspnea, chest tightness and cough, which is life-threatening (Boulet et al., 2019). Studies have reported that the presence of asthma can increase the production of reactive oxygen species, and the antioxidant system in the lungs isn’t sufficient to alleviate it (Liu et al., 2022; Wang et al., 2022). Thus, asthma may cause the body oxidative stress, which aggravates airway inflammation and airway hyperresponsiveness (Michaeloudes et al., 2022).

Antioxidants can counteract the damaging effects of oxidative stress and play a crucial role in maintaining respiratory health (Nadeem et al., 2003). Vitamin A (vit A) is a non-enzymatic antioxidant derived from plants (carotenoids) or animal origin (retinol) and it plays an important role in lung development, respiratory epithelium and immune system (Trivillin et al., 2022). Vit A plays a central role in asthma by promoting T cell proliferation and prolonging their survival, enhancing antigen-presenting capacity and promoting Th2 cell response (Malinovschi et al., 2011). However, the relationship between dietary vit A and risk of asthma in humans is unclear. A cross-sectional study reported that serum vit A was negatively correlated with the severity of asthma in children (Chérot-Kornobis et al., 2011). In adults, serum retinol was inversely associated with subsequent airway obstruction (Morabia et al., 1990), however it has recently been found that there was a positive correlation between vit A intake and asthma (Mai et al., 2013). A follow-up trial in a vit A-deficient population showed that early gestational vit A supplementation improved lung function in offspring (Checkley et al., 2010), with no effect on subsequent asthma risk (Checkley et al., 2011).

Consequently, there is an urgent need for evidence-based information obtained from systematic synthesis of studies on the association between vit A and asthma risk for the primary prevention of asthma onset. The aim of this systematic review was to synthetize the available scientific evidence on the association of vit A and asthma risk, thus supporting or preventing the use of vit A.

## Materials and methods

This study doesn’t require ethical approval and informed consent because it is a systematic review and meta-analysis of previously published literature and doesn’t address ethics or patient privacy. Our study was analyzed and reported according to the Preferred Reporting Items for Systematic Reviews and Meta-Analyses (PRISMA) (Moher et al., 2009). The study protocol was registered and approved in the International Prospective Register of Systematic Reviews (PROSPERO) under the code CRD42022358930.

### Search strategy

Two reviewers independently performed a comprehensive literature search in four electronic databases, including PubMed, Web of Science, Embase and the Cochrane Library. All databases were searched from inception to November 2022. The following MeSH terms and keywords were searched (“vitamin A” OR “vit A” OR “aquasol A” OR “retinol” OR “all-trans-retinol” OR “vitamin A1” OR “11-cis-retinol”) AND (“asthma” OR “asthmas” OR “bronchial asthma”). References within the identified articles were manually examined to identify other potentially eligible studies. The details of the search strategy were provided in Supplementary Material S1.

### Inclusion and exclusion criteria

The following criteria were predefined for inclusion of a study: 1) the original observational or interventional study reported the relationship between vit A and asthma risk; ii) the study provided the serum vit A or vit A intake levels, relative risks (RRs) or odds ratios (ORs) and 95% confidence intervals (CIs); iii) cases were diagnosed with asthma (informed by clinician). The exclusion criteria were as follows: i) the cases weren’t only asthma patients, but also patients with wheezing, atopic dermatitis, allergic rhinoconjunctivitis or other allergic disease, resulting in the inability to independently obtain the association of vit A and asthma; ii) studies with insufficient data; iii) reviews or meta-analyses, conference abstract, and study protocols.

### Data extraction and quality assessment

Screening of studies, selection, exclusion, and data extraction were performed by two reviewers independently. Any disagreements were discussed and reached a consensus. We extracted the following information from case-control and cohort studies: first author, publication year, study location and design, participants, age, vitamin measurements, statistical methods and outcomes of interest.

Case-control and cohort studies were assessed using the Newcastle-Ottawa scale (NOS) (Wells et al., 2014) consisting of three domains: 1) selection of subjects, 2) comparability of groups, and 3) assessment of outcome. A score of 0–9 was allocated to each relevant study. While the NOS has no established thresholds, we considered the quality of each study as low (0–3 score), moderate (4–6 score), or high (7–9 score) (Chung et al., 2021).

### Statistical analysis

Statistical analyses were performed with R software Version 4.1.2 and STATA Version 12.0 (StataCorp, College Station, TX, United States). Data of dichotomous outcomes were pooled using the risk ratio (RR) or odds ratio (OR) and presented as the 95% confidence interval (CI). Continuous outcomes were pooled using the standard mean difference (SMD) and 95%CI. Heterogeneity was assessed statistically by using the Cochran’s Q test, I^2^ and Tau^2^ statistic and 95% prediction interval (PI) (Bowden et al., 2011; IntHout et al., 2016). When *p* > 0.1 or I^2^ ≤ 50%, the results of the associated studies were considered to have no significant heterogeneity, and a fixed-effects model was utilized. When *p* ≤ 0.1 or I^2^ > 50%, it was considered that there was heterogeneity in the results of the included studies, and a random-effects model was selected (Higgins and Thompson, 2002). The presence of publication bias was assessed with the Begg’s adjusted rank correlation test and the Egger’s regression asymmetry test (Begg and Mazumdar, 1994; Sterne and Egger, 2001). Sensitivity analysis was conducted to explore the possible sources of heterogeneity.

### Trial sequential analysis

We performed a trial sequential analysis (TSA) to assess if the available evidence is up to the required information size (RIS) for robust conclusion (Wetterslev et al., 2017). For continuous outcomes, the trial sequential analysis was performed using TSA v0.9.5.10 Beta software (www.ctu.dk/tsa). In the present trial sequential analysis, we estimated the RIS and built O’ Brien-Fleming α-spending boundaries by using type I error of 5% and type II error of 20%, which were two-side values. If the cumulative Z-curve crossed the trial sequential monitoring boundary or RIS boundary, no further trials were considered to be needed and firm evidence was obtained.

## Results

### Literature search

**FIGURE 1:**
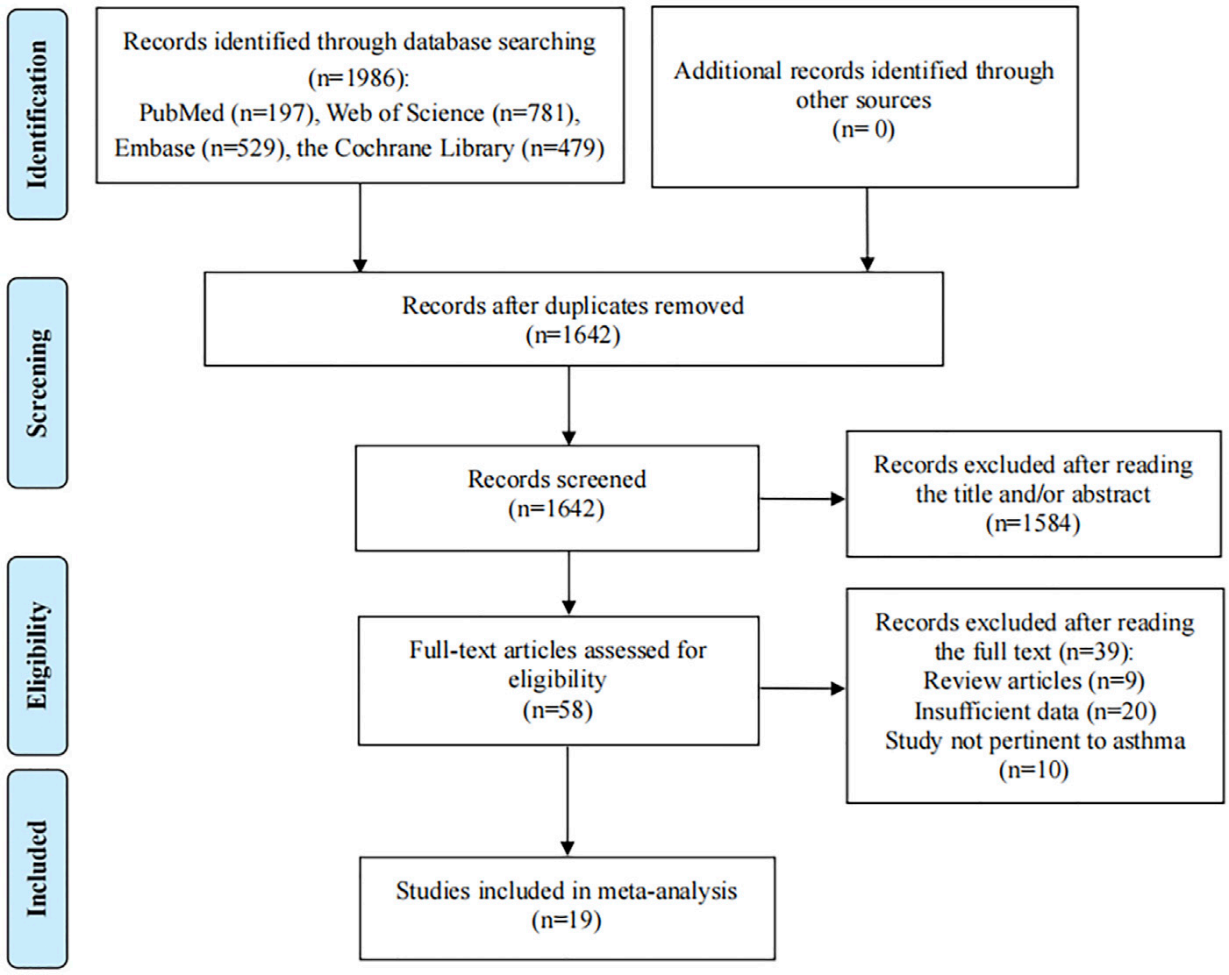
Flow diagram of the process of selection of articles.

The initial search identified 1986 relevant studies. After 344 duplicate studies were excluded, 1,642 articles remained. Then 1,584 articles were excluded after screening the titles and abstracts according to the eligibility criteria and 58 potential articles were reviewed for full text. After reading the full text, 39 articles didn’t meet the inclusion criteria: nine studies were review articles; 20 articles provided insufficient data; 10 articles reported wheezing or other allergic disease. Finally, 19 eligible studies were included in the present meta-analysis (Figure 1) (Hijazi et al., 2000; Arora et al., 2002; Harik-Khan et al., 2004; Rubin et al., 2004; Mizuno et al., 2006; Murray et al., 2006; Al Senaidy, 2009; Rosenlund et al., 2012; Nakamura et al., 2013; Maslova et al., 2014; Lee et al., 2015; Kim et al., 2016; Bishopp et al., 2017; Kodama et al., 2017; Bai and Dai, 2018; Parr et al., 2018; Muhsen et al., 2019; Talaei et al., 2021; Podlecka et al., 2022).

### Characteristics and quality assessment of the included studies

16 case-control studies and three cohort studies were included in our meta-analysis. Studies were performed in Asia, Europe and America, respectively. The participants were asthmatic patients or non-asthmatics, including children, adolescents and adults. We extracted the outcomes of serum vit A and vit A intake levels in the asthma and healthy group. As the studies of Bai and Bishopp showed, the study population was respectively divided into Severe, Moderate, Mild, Control groups (Bai and Dai, 2018) and Severe asthma, Mild asthma, Healthy controls (Bishopp et al., 2017). We extracted the serum vitamin A levels of “Severe vs. Control,” “Moderate vs. Control,” “Mild vs. Control” and they were represented by “Bai 2018a,” “Bai 2018b” and “Bai 2018c” respectively. The OR or RR and their 95% CI were extracted to evaluate the association between vit A and asthma (Table 1). Three case-control studies were considered as moderate quality. The other 13 case-control and three cohort studies were assessed as high quality, because the study design had been described in detail (Supplementary Material S2).

**TABLE 1:** Characteristics of observational studies included in the meta-analysis.

Study	Location	Design	Population size	Population groups	Age, years	Vit A measurements	Statistical methods	Outcomes	Adjustments
Al Senaidy 2009	Saudi Arabia	Case-control	Case: 433 Control: 537	Children and adolescents	6–18	Reverse phase HPLC	NR	Serum vit A levels	No adjustments
Arora 2002	India	Case-control	Case: 35 Control: 29	Children	2–12	HPLC	Student’s t-test	Serum vit A levels	No adjustments
Bai 2018	China	Case-control	Case: 117 Control: 129	Children	Case: 8.52 ± 2.37 Control: 8.93 ± 2.76	Chemiluminescence immunoassay	Joint hypotheses test	Serum vit A levels	No adjustments
Bishopp 2017	England	Case-control	Case: 45 Control: 15	Adults	18–60	HPLC	One-way analysis of variance	Serum vit A levels	No adjustments
Harik-Khan 2004	United States	Case-control	Case: 397 Control: 3,696	Children and adolescents	6–17	isocratic HPLC	Student’s t-test	Serum vit A levels, association between serum vit A levels and asthma in children	Age, gender, household size, BMI, household head educational status, household head gender, household head employment status, diagnosis of asthma or hay fever in at least one parent, race, the presence of at least one smoker in the household, and serum levels of the antioxidant vitamins C and E and the carotenoids: α-carotene, β-carotene, β-cryptoxanthin, lutein/zeaxanthin, and lycopene
Hijazi 2000	Saudi Arabia	Case-control	Case: 114 Control: 202	Children	12 ± 1	Questionnaire enquiring	Student’s t-test	Levels of vit A intake	No adjustments
Kim 2016	Korea	Case-control	Case: 182 Control: 2,858	Children	4–13	Complete 24-h recall method	Multiple logistic regression	Associations between vit A intake and child asthma	Age, sex, BMI, the number of household members, income level, and region of residence
Kodama 2017	Japan	Case-control	Case: 15 Control: 28	Elderly people	≥40	HPLC	One-way analysis of variance	Serum vit A levels	No adjustments
Lee 2015	Korea	Case-control	Case: 110 Control: 1,082	Children	7–12	ISAAC questionnaire and FFQ	Multiple logistic regression	Associations between vit A intake and child asthma	Age, sex, BMI, parental history of asthma, exposure to environmental tobacco smoke, maternal education, household income, and log-transformed total energy intake
Maslova 2014	Denmark	Cohort	28,399 participants	Pregnancy women and children	18 months and 7 years	FFQ and self-administered questionnaire	Log-binomial models	Associations between maternal total vit A intake in mid-pregnancy and child asthma at 7 years	Maternal age, socio-economic status, parity, pre-pregnancy BMI, maternal physical activity, maternal smoking during pregnancy, breast-feeding duration, child sex, maternal history of asthma, maternal history of allergies, paternal history of asthma, paternal history of allergies, season of last menstrual period, and energy, fruit intake, vegetable intake, and intake of fish fatty acids, folic acid, vitamins D and C, Se and Zn
Mizuno 2006	Japan	Case-control	Case: 26 Control: 25	Children	mean age, 5.5	NR	Mann-Whitney U-test	Serum vit A levels	Age-sex matched controls
Muhsen 2019	Iraq	Case-control	Case: 80 Control: 30	Children	1–13	ELISA	Student’s t-test	Serum vit A levels	Age- and sex-matched controls
Murray 2006	England	Case-control	Case: 37 Control: 37	Children	mean age, 4.4	NR	Paired *t*-test	Levels of vit A intake	Age-matched, sex-matched controls
Nakamura 2013	Japan	Case-control	Case: 37 Control: 415	Pre-school children	3–6	3 days dietary record	Logistic regression	Associations between vit A intake and asthma among pre-school children	Child’s age, sex, and BMI, breast-feeding, child’s history of food allergy, mother’s age, parental history of allergy, maternal education level, the number of siblings and household smoking
Parr 2018	Norwegian	Cohort	600 cases and 12,346 total	Mother and Child	7	FFQ	Log binomial regression model	Association between total vit A intake in pregnancy and current asthma at age 7 years	Maternal total intakes of vitamins D, vitamin E, vitamin C, folate, sum of n-3 fatty acids, total energy intake, age at delivery, parity, education, prepregnancy BMI, history of asthma, history of atopy, smoking in pregnancy, birth weight and prematurity
Podlecka 2022	Poland	Case-control	Case: 40 Control: 40	Children	9–12	HPLC	Logistic regression models	Association between serum vit A levels and asthma in children	Mother’s age, mother’s education, income
Rosenlund 2012	Sweden	Cohort	2,442 children at the age of 8	Children	8	FFQ	Logistic regression	Associations between intake of vit A and asthma among 8-year-old children	Sex, parental socioeconomic status, maternal smoking during pregnancy and/or at baseline, iso-BMI, parental history of allergic disease and vitamin supplementation
Rubin 2004	United States	Case-control	Case: 415 Control: 5,305	Children and adolescents	4–16	isocratic HPLC	NR	Serum vit A levels, association between serum vit A levels and asthma in children	Age, sex, race, BMI, passive smoke exposure, active smoke exposure, parental history of asthma and/or hay fever, urban environment, household crowding, years of education of the head of household, childhood history of hayfever, and avoidance of pet ownership caused by allergy
Talaei 2021	England	Case-control	Case: 93 Control: 1,024	Children	11 or 14	FFQ	Logistic regression models	Associations between vit A intake and child asthma	Sex, total energy intake, maternal education, housing tenure at birth, financial difficulty during pregnancy, maternal ethnicity, maternal history of atopic disease, paternal history of atopic disease, maternal smoking, older sibling, younger sibling, supplement use and season when the FFQ was completed

Vit A, vitamin A; ISAAC, international study of asthma and allergies in childhood; FFQ, the food frequency questionnaire; ELISA, enzyme-linked immunosorbent assay kit; HPLC, high performance liquid chromatography.

### Comparison of serum vit A and vit A intake levels between asthmatic and healthy groups

**FIGURE 2:**
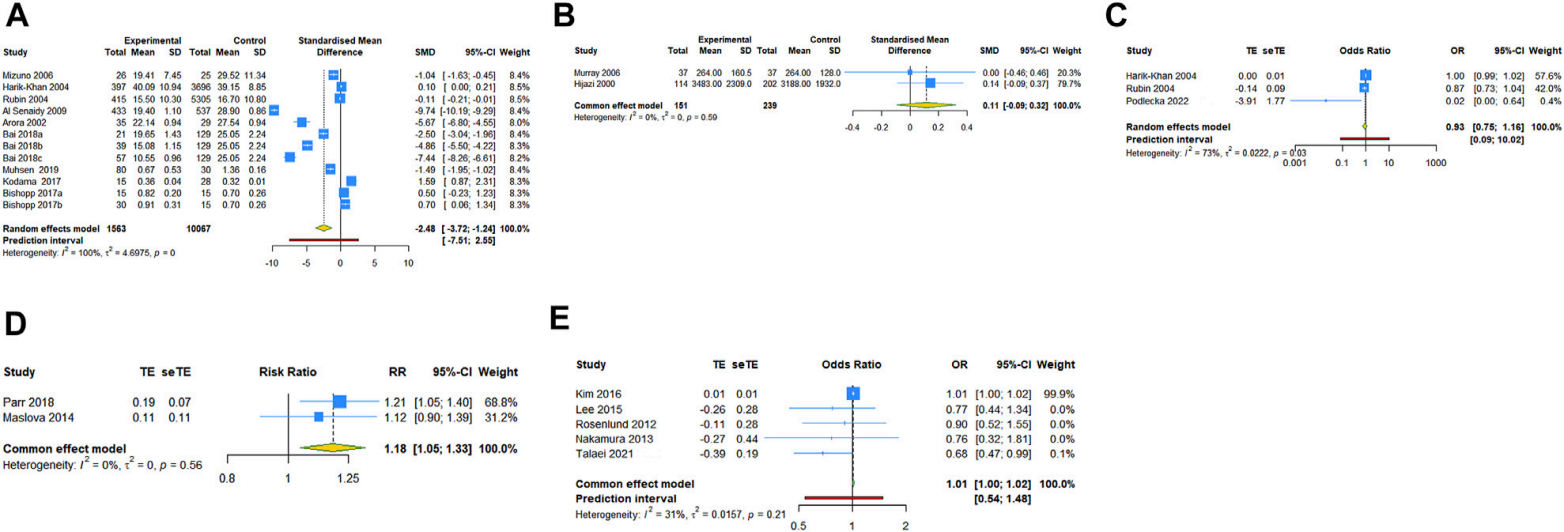
Forest plot of vit A and asthma **(A)** comparison of serum vit A levels between asthma and control groups; **(B)** comparison of vit A intake levels between asthma and control groups; **(C)** association between serum vit A levels and asthma risk; **(D)** association between vit A intake in pregnancy and current asthma at age 7 years; **(E)** association between vit A intake and asthma risk in children. TE, treatment effect; seTE, standard error of treatment effect.

12 case-control studies reported the serum vit A concentrations in asthmatic and healthy groups. Our analysis with a random-effect model showed that the levels of serum vit A in asthmatic patients was lower than that in healthy controls (SMD = −2.479, 95% CI: −3.719, −1.239, 95% PI: −7.510, 2.552; I^2^ = 99.5%, Tau^2^ = 4.6975) (Table 2; Figure 2A). The meta-analysis of two included case-control studies showed no difference in vit A intake levels between asthmatic patients and healthy controls (SMD = .113, 95% CI: −.092, .318; I^2^ = 0, Tau^2^ = 0) (Table 2; Figure 2B).

**TABLE 2:** Meta-analysis in the association of vitamin A and asthma.

Outcomes	Number of studies	Meta-analysis				Heterogeneity	
		SMD/OR/RR	95% CI	*p*-Value	95% PI	I^2^, Tau^2^	*p*-Value
Comparison of serum vitamin A levels between asthma and control groups	12	−2.479	−3.719, −1.239	<.001	−7.510, 2.552	99.5%, 4.6975	<.001
Comparison of vitamin A intake levels between asthma and control groups	2	.113	−.092, .318	.280	-	0, 0	.586
Association between serum vitamin A levels and asthma risk (OR)	3	.931	.745, 1.162	.524	.086, 10.022	72.8%, .0222	.025
Association between vitamin A intake in pregnancy and current asthma risk at age 7 years (RR)	2	1.181	1.048, 1.331	.006	–	0, 0	.557
Association between vitamin A intake and asthma risk in children (OR)	5	1.010	1.000, 1.020	.060	.542, 1.480	31.3%, .0157	.213

### The association between vit A and asthma risk

Three case-control studies reported the association between serum vit A levels and asthma risk. The result with a random-effect model showed that there was no significant relationship between serum vit A concentrations and asthma risk (OR = 0.931, 95% CI: 0.745, 1.162; I^2^ = 72.8%, Tau^2^ = 0.0222) (Table 2; Figure 2C). Two cohort studies reported the association between vit A intake in pregnancy and current asthma risk at age 7 years, and the pooled analysis showed that relatively higher vit A intake in pregnancy was associated with an increased risk of current asthma at 7 years of age (RR = 1.181, 95% CI: 1.048, 1.331; I^2^ = 0, Tau^2^ = 0) (Table 2; Figure 2D). However, no significant relationship was observed in children between vit A intake and asthma risk (OR = 1.010, 95% CI: 1.000, 1.020, 95% PI: .542, 1.480; I^2^ = 31.3%, Tau^2^ = .0157) (Table 2; Figure 2E).

### Subgroup analyses

**FIGURE 3:**
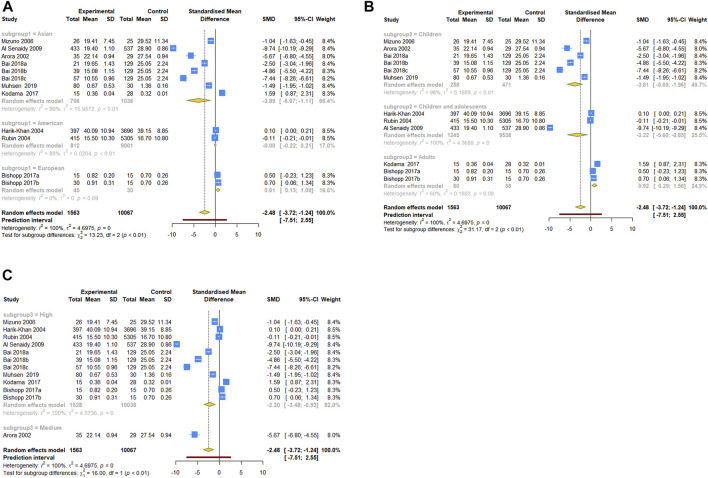
Forest plot of subgroup analysis of comparison for serum vit A levels between asthma and control groups. **(A)** Subgrouped by ethnicity; **(B)** subgrouped by population; **(C)** subgrouped by quality score.

**FIGURE 4:**
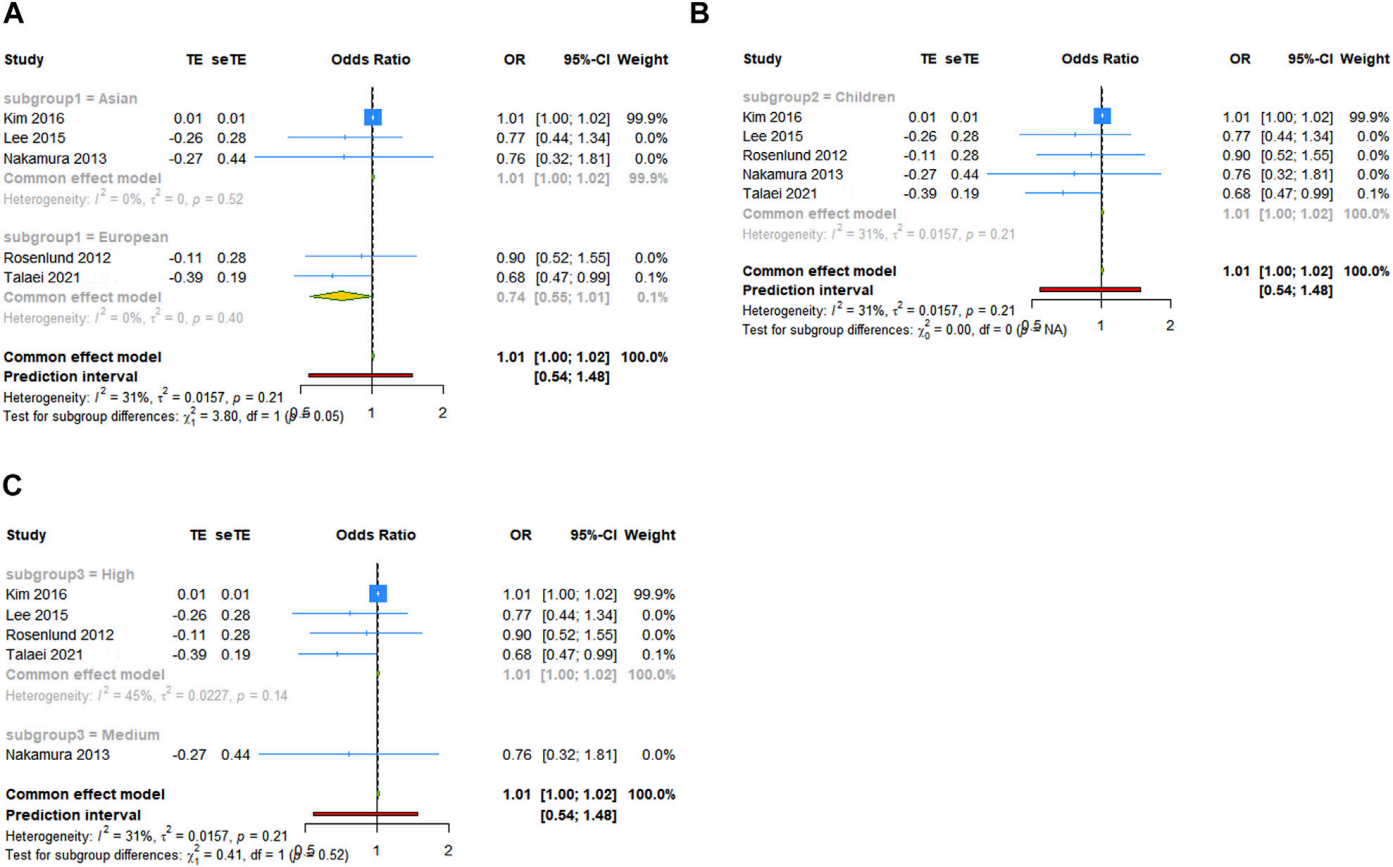
Forest plot of subgroup analysis of association between vit A intake and asthma risk in children. **(A)** Subgrouped by ethnicity; **(B)** subgrouped by population; **(C)** subgrouped by quality score. TE, treatment effect; seTE, standard error of treatment effect.

For the comparison of serum vit A levels between asthmatic and healthy groups, we conducted a subgroup analysis by ethnicity, population or quality score. In a subgroup analysis conducted by ethnicity, we obtained significant result that the serum vit A concentration of asthmatic patients was lower than that of healthy controls in Asian populations (SMD = −3.891, 95% CI: −6.671, −1.112, 95% PI: −14.263, 6.481; I^2^ = 99.4%, Tau^2^ = 15.9572). Similar results were observed in subgroups of children, children and adolescents or high-quality studies. However, patients with asthma had higher serum vit A levels than healthy controls in European populations (SMD = .615, 95% CI: .135, 1.095; I^2^ = 0, Tau^2^ = 0) or adults (SMD = .925, 95% CI: .290, 1.559, 95% PI: −5.967, 7.816; I^2^ = 60.2%, Tau^2^ = 0.1893) (Table 3; Figure 3). No significant association in subgroup analysis was obtained between vit A intake and asthma risk in children (Table 3; Figure 4).

**TABLE 3:** Subgroup analysis in the association of vitamin A and asthma.

Outcomes and stratified analyses	Number of studies	Meta-analysis				Heterogeneity	
		SMD/OR	95%CI	*p*-Value	95% PI	I^2^, Tau^2^	*p*-Value
Comparison of serum vitamin A levels between asthma and control groups							
Ethnicity							
Asian	8	−3.891	−6.671, −1.112	.006	−14.263, 6.481	99.4%, 15.9572	<.001
American	2	−.004	−.215, .206	.968	–	88.3%, .0204	.003
European	2	.615	.135, 1.095	.012	–	0, 0	.693
Population							
Children	6	−3.806	−5.650, −1.963	<.001	−10.637, 3.025	98.0%, 5.1689	<.001
Children and adolescents	3	−3.225	−5.596, −.855	.008	−33.909, 27.459	99.9%, 4.3689	<.001
Adults	3	.925	.290, 1.559	.004	−5.967, 7.816	60.2%, .1893	.081
Quality score							
High	11	−2.202	−3.478, −.927	<.001	−7.259, 2.855	99.6%, 4.5736	<.001
Medium	1	−5.675	−6.801, −4.548	<.001			
Association between vitamin A intake and asthma risk in children (OR)							
Ethnicity							
Asian	3	1.010	1.000, 1.020	.052	.947, 1.077	0, 0	.516
European	2	.744	.547, 1.011	.059	–	0, 0	.404
Population							
Children	5	1.010	1.000, 1.020	.060	.542, 1.480	31.3%, .0157	.213
Quality score							
High	4	1.010	1.000, 1.020	.059	.396, 1.986	44.6%, .0277	.144
Medium	1	.760	.320, 1.808	.535			

### Trial sequential analysis results

**FIGURE 5:**
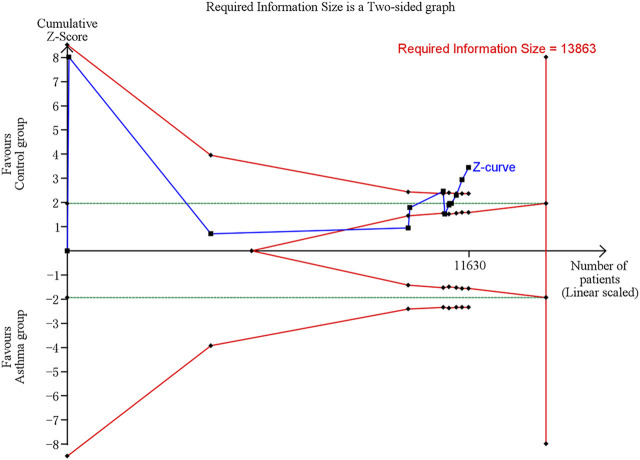
Trial sequential analysis (TSA) of comparison of serum vit A levels between asthma and control groups. Uppermost and lowermost red curves represent trial sequential monitoring boundary lines for benefit and harm, respectively. Horizontal green lines represent the conventional boundaries for statistical significance. Inner red lines represent the futility boundary.

In trial sequential analysis of the comparison of serum vit A levels between asthma and control groups, we estimated a required sample size of 13,863, and the cumulative Z-curve significantly crossed the conventional monitoring boundary and trial sequential monitoring boundary, but didn’t cross the RIS boundary, further suggesting that the pooled result is less likely to be a random finding owing to a lack of power or multiple testing if bias could be ignored (Figure 5).

### Publication bias and sensitivity analysis

Given the limited number of studies included in the pooled analysis, we performed publication bias test and sensitivity analysis for the pooled result which included 12 studies. Begg’s test and Egger’s test were conducted to evaluate the publication bias and the results revealed that no significant publication bias existed among our studies (Begg’s test: *p* = 0.451, Egger’s test: *p* = 0.452). The funnel plot was shown in Supplementary Figure S1 (Supplementary Material S3). To assess whether the pooled results were affected by a single study, we conducted a sensitivity analysis by computing the pooled SMDs and the corresponding 95% CIs after individual studies were omitted. The sensitivity analysis indicated that Harik-Khan, Rubin or Al Senaidy’s article may be the cause of high heterogeneity (Supplementary Files S3).

## Discussion

Asthma is characterized by chronic airway inflammation and many features of asthma are caused by the release of pro-inflammatory mediators induced by oxidants (Caramori and Papi, 2004). Oxidative stress leads to inflammation and tissue damage of the respiratory system, which subsequently results in activation to the immune system (Bowler and Crapo, 2002). Antioxidants protect the respiratory tract by preventing oxidative DNA damage (Marmsjö et al., 2009). Thus, it has been reported that decreased antioxidant intake due to dietary changes leads to attenuate antioxidant defenses in the lung and increased susceptibility to airway inflammation and asthma (Lazarus et al., 2007; Marmsjö et al*.*, 2009). Vit A is an antioxidant, which may play a major role in the respiratory tract through physiological quenching of singlet oxygen and inhibition of free radical chain reaction (Harik-Khan et al., 2004; Wagner et al., 2008). Epidemiological studies have investigated the relationship of dietary vit A intake to asthma, however, the association between asthma and vit A status isn’t fully understood. Hence, the primary objective of present meta-analysis was to understand the relationship between vit A status and asthma and to clarify the effect of dietary vit A intake on asthma.

Vit A is primarily stored in the liver, which closely regulates the circulating retinol levels, and serum retinol levels do not decline until the liver is almost depleted (Timoneda et al., 2018). Serum vit A biomarker concentrations reflect the combined effect of dietary intake, bioavailability and metabolism. Our pooled analysis showed that serum vit A concentrations were lower in patients with asthma than in healthy controls. The triggers of asthma can result in the recruitment and activation of airway inflammatory cells, produce more oxygen-derived free radicals, and lead to oxidative damage (Wood et al., 2003). Increased systemic free radical production is a characteristic of chronic airway inflammation (Vural et al., 2005). Retinoic acid is an active metabolite of vit A, which can influence the maintenance, development, differentiation of lung epithelial cells, thereby reducing eosinophilic airway inflammation (Gray et al., 2001). Airway inflammation leads to an increase in lipid peroxides, providing cogent evidence that cell membrane damage may induce cell dysfunction and lead to exacerbation of airway obstruction (Al Senaidy, 2009). Therefore, we speculate that the free radical accumulation is associated with defective mobilization of stored retinol. The decrease of vit A levels in patients with asthma may be caused by increased utilization of this antioxidant in the presence of excess free radicals. However, no significant association was shown between serum vit A concentration and asthma risk in our study. Consequently, it is unclear whether low vit A level is either a main cause related to the pathogenesis of asthmatic condition, or a secondary event caused by the inflammatory process of asthma.

Few human studies, to our knowledge, have assessed the risk of child asthma in relation to pregnancy intake of vit A. Many aspects of maternal-fetal transfer of retinoic acid and carotenoid, their metabolism in developing tissues, and the control of homeostasis in the case of excessive maternal dietary vit A intake are still poorly known (Parr et al., 2018). Through retinoic acid, vit A is known to regulate the function of immune cells (Hammerschmidt et al., 2008). For example, a significant body of evidence shows that retinoic acid potently regulates T cell activity, but may be either pro- or anti-inflammatory depending on conditions (Takeuchi et al., 2013; Rampal et al., 2016). It has been reported in animal studies that vit A deficiency decreased the development of experimental asthma, indicating that vit A deficiency might skew towards Th1 cell-mediated responses and away from Th2 cell-mediated responses that induces asthma (Cantorna et al., 1995; Schuster et al., 2008). Nevertheless, human studies showed that vit A supplementation in pregnancy increased Th1 cell-mediated responses and skewed away from Th2 cell-mediated responses, suggesting that the skewed *in utero* milieu can decrease the risk of asthma in children (Holgate, 2004; Cox et al., 2006). Our analysis of cohort studies showed that relatively higher vit A intake during pregnancy was associated with an increased risk of asthma in children at age 7 years. As the two cohort studies included in the pooled analysis were from Norway and Denmark, we attribute the association in our study to the high retinol content of Norwegian cod liver oil and the traditional diet rich in vit A (Parr et al., 2018). We speculated that an increased intake of vit A during pregnancy, based on a diet naturally high in vit A, placed women at risk of vit A excess, thus increasing susceptibility to asthma in children.

Subgroup analysis revealed that in Asian populations, the serum vit A concentration in patients with asthma was lower than that in healthy controls, while an opposite result was obtained in European populations. The disparity of results for different ethnicity might be explained by genetic variance, culture and dietary habits, socioeconomic status, healthcare system, and so forth (Kodama et al., 2017). A recent study showed that genetic polymorphisms may be contributing to the vit A deficiency status differences between ethnic groups (Suzuki et al., 2021). The data suggests a need for more studies outside Asia (e.g., America, Europe) to detect differences, given that eight of the twelve studies were in Asia. Furthermore, in adults, serum vit A levels were higher in asthmatics than in healthy controls. A plausible explanation for the finding is the effect of obesity (Bishopp et al., 2017). We found that the asthma groups had a higher mean BMI than the healthy control groups in original articles. Obesity affects numerous metabolic pathways, including vit A, although the exact nature of its effects has not been determined (Hijazi et al., 2000). Previous studies have reported that serum vit A levels of obese mice increased, and with weight loss, the serum vit A levels returned to the normal range (Trasino and Gudas, 2015). It is reported that obese individuals have higher levels of systemic oxidative stress, and hypertrophic adipose tissue produces proinflammatory cytokines and adipokines, leading to metabolic inflammation (Ford et al., 2004; Urushidate et al., 2010). Obesity is a risk factor for asthma, but the definite interaction between obesity and asthma is still not clear (Hoffmann et al., 2007).

Our study has several limitations. First, any dietary assessment method is subject to measurement error. The use of both dietary recall instrument and food frequency questionnaires to estimate dietary vit A intake provided relatively imprecise quantitative estimates. Second, assessing the dietary habits of vit A in young children seems difficult, because they often eat little food and are unable to adequately report their vit intake. In practice, parents are unlikely to always be good surrogate reporters for their children’s diet. Third, studies in humans are limited by potential toxic effects and the lack of viable biomarkers to assess appropriate or subtoxic status. Thus, no randomized controlled study was included in present study to evaluate the effect of vit A supplementation on asthma. In addition, more relevant cohort studies are needed to be included in our pooled analysis to further enhance the stability of the result.

## Conclusion

In conclusion, our meta-analysis confirms that serum vit A levels are lower in patients with asthma than in healthy controls, particularly in Asian and non-adult subpopulations. Relatively higher vit A intake during pregnancy is associated with an increased risk of asthma at age 7 years. There is no significant correlation between vit A intake and asthma risk in children, nor between serum vit A levels and asthma risk. Our analysis also suggests that the interaction of vit A and asthma may depend on age, ethnicity, or other factors, or be obscured in heterogeneous populations. Therefore, more studies are needed to further explore the association of vit A and asthma.

## Data Availability

The original contributions presented in the study are included in the article/Supplementary Material, further inquiries can be directed to the corresponding authors.
