# Supplementary material for: Association between vitamin A and asthma: A meta-analysis with trial sequential analysis
Source: Front Pharmacol. 2023 Jan 30;14:1100002. doi: 10.3389/fphar.2023.1100002 (PMC9922757; doi:10.3389/fphar.2023.1100002)
Supplement: Supplementary file 1 [file Table1.DOCX]

Pubmed 197

((vitamin A) OR (vit A) OR (aquasol A) OR (retinol) OR (all-trans-retinol) OR (vitamin A1) OR (11-cis-retinol)) AND ((asthma) OR (asthmas) OR (bronchial asthma))

Web of science 781

(TS=(vitamin A) OR TS=(vit A) OR TS=(aquasol A) OR TS=(retinol) OR TS=(all-trans-retinol) OR TS=(vitamin A1) OR TS=(11-cis-retinol)) AND (TS=(asthma) OR TS=(asthmas) OR TS=(bronchial asthma)) with (Review or Meeting or Abstract or Case Report or Letter)[Excluding-Type of literature] and (Asthma)[MeSH terms]).

Embase 529

('vitamin a'/exp OR 'vitamin a') AND ('asthma'/exp OR 'asthma')

The cochrane library 479

((vitamin A) OR (aquasol A) OR (retinol) OR (all-trans-retinol) OR (vitamin A1)) - in All Text AND ((asthma) OR (asthmas) OR (bronchial asthma)) - in All Text - (Word variations have been searched)
