# Supplementary material for: Association between vitamin A and asthma: A meta-analysis with trial sequential analysis
Source: Front Pharmacol. 2023 Jan 30;14:1100002. doi: 10.3389/fphar.2023.1100002 (PMC9922757; doi:10.3389/fphar.2023.1100002)
Supplement: Supplementary file 2 [file Table2.DOCX]

| **Table S1** Case-control studies bias assessment using the Newcastle-Ottawa Scale (NOS). | | | | | | | | | | | |
| --- | --- | --- | --- | --- | --- | --- | --- | --- | --- | --- | --- |
| Study | SELECTION | | | |  | COMPARABILITY |  | EXPOSURE | | | Total score |
|  | Is the case definition adequate? | Representativeness of the cases? | Selection of controls? | Definition of controls? |  | Comparability of cases and controls of design or analysis? |  | Ascertainment of exposure? | Same method of ascertainment for cases | Non-response rate? |  |
|  |  |  |  |  |  |  |  |  |  |  |  |
| Mizuno 2006 | 1 | 1 | 1 | 1 |  | 1 |  | 1 | 1 | 1 | 8 |
| Talaei 2021 | 1 | 1 | 1 | 1 |  | 2 |  | 1 | 1 | 1 | 9 |
| Murray 2006 | 1 | 1 | 1 | 1 |  | 2 |  | 1 | 1 | 0 | 8 |
| Harik-Khan 2004 | 1 | 1 | 1 | 1 |  | 0 |  | 1 | 1 | 1 | 7 |
| Podlecka 2022 | 1 | 1 | 1 | 1 |  | 1 |  | 1 | 1 | 1 | 8 |
| Rubin 2004 | 1 | 1 | 1 | 1 |  | 0 |  | 1 | 1 | 1 | 7 |
| Hijazi 2000 | 0 | 1 | 1 | 0 |  | 1 |  | 1 | 1 | 1 | 6 |
| Arora 2002 | 1 | 0 | 1 | 1 |  | 1 |  | 1 | 1 | 0 | 6 |
| Al Senaidy 2009 | 1 | 1 | 1 | 1 |  | 1 |  | 1 | 1 | 0 | 7 |
| Bai 2018 | 1 | 1 | 1 | 1 |  | 1 |  | 1 | 1 | 1 | 8 |
| Kim 2016 | 1 | 1 | 1 | 1 |  | 1 |  | 1 | 1 | 1 | 8 |
| Lee 2015 | 1 | 1 | 1 | 1 |  | 0 |  | 1 | 1 | 1 | 7 |
| Muhsen 2019 | 1 | 0 | 1 | 1 |  | 2 |  | 1 | 1 | 0 | 7 |
| Kodama 2017 | 1 | 1 | 1 | 1 |  | 1 |  | 1 | 1 | 1 | 8 |
| Nakamura 2013 | 1 | 1 | 1 | 1 |  | 0 |  | 1 | 1 | 0 | 6 |
| Bishopp 2017 | 1 | 0 | 1 | 1 |  | 1 |  | 1 | 1 | 1 | 7 |

| **Table S2** Cohort studies bias assessment using the Newcastle-Ottawa Scale (NOS). | | | | | | | | | | | | |
| --- | --- | --- | --- | --- | --- | --- | --- | --- | --- | --- | --- | --- |
| Study | Selection | | | |  | Comparability | |  | Assessment of outcome | | | Total score |
|  | Representativeness of exposure arm(s) | Selection of the comparative arm(s) | Origin of exposure source | Demonstration that outcome of interest was not present at start of study |  | Studies controlling the most important factors | Studies controlling the other main factors |  | Assessment of outcome with independency | Adequacy of follow-up length | Lost to follow-up acceptable |  |
|  |  |  |  |  |  |  |  |  |  |  |  |  |
| Parr 2018 | 1 | 1 | 1 | 1 |  | 1 | 0 |  | 1 | 1 | 1 | 8 |
| Maslova 2014 | 1 | 1 | 1 | 1 |  | 1 | 0 |  | 1 | 1 | 1 | 8 |
| Rosenlund 2012 | 1 | 1 | 1 | 1 |  | 1 | 0 |  | 1 | 1 | 1 | 8 |
