# Supplementary material for: Association between vitamin A and asthma: A meta-analysis with trial sequential analysis
Source: Front Pharmacol. 2023 Jan 30;14:1100002. doi: 10.3389/fphar.2023.1100002 (PMC9922757; doi:10.3389/fphar.2023.1100002)

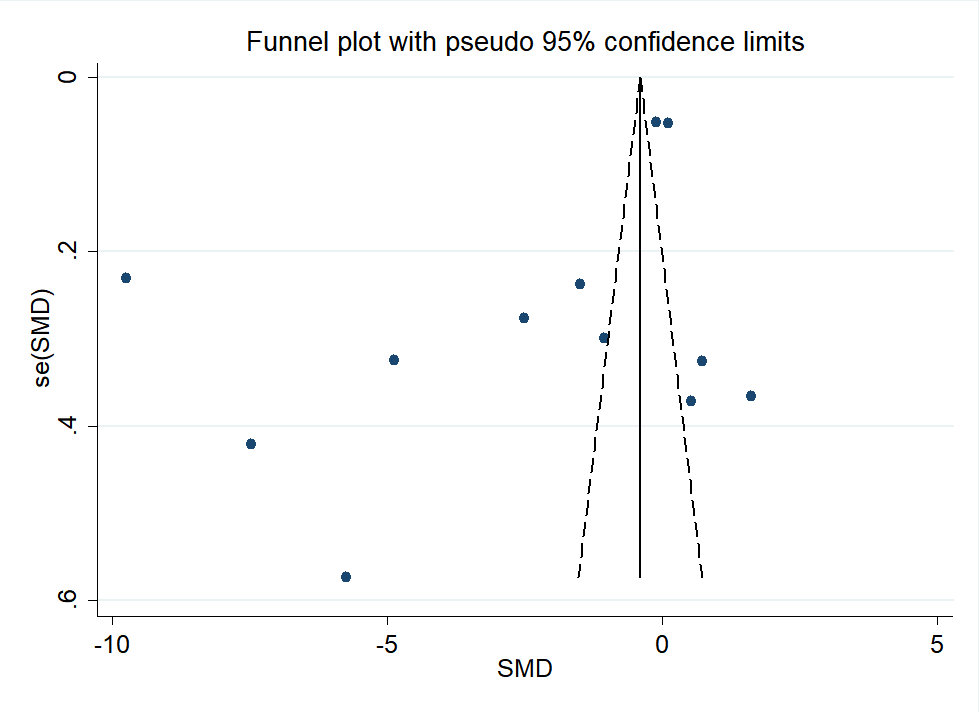


**Figure S1** Funnel plot of publication bias on the comparison of serum vit A levels between asthma and control groups.

**Figure S2** Sensitivity analysis on the comparison of serum vit A levels between asthma and control groups.


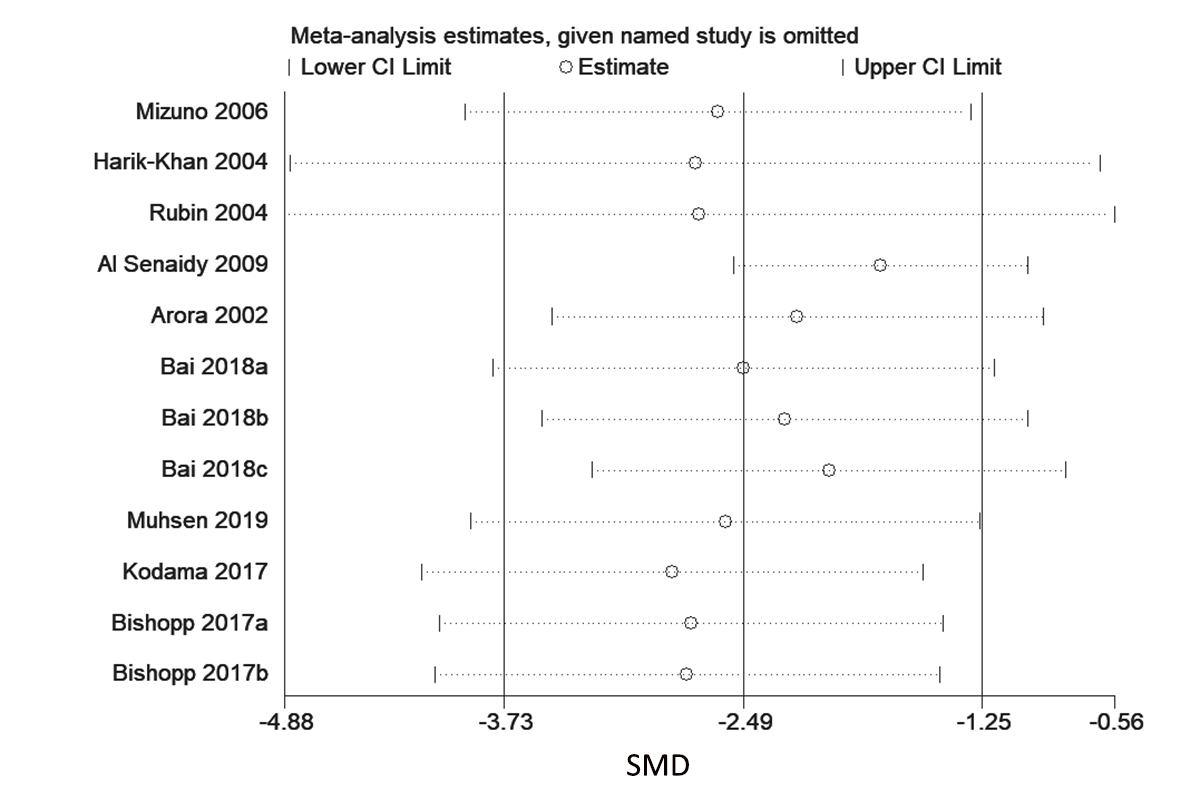

Supplement: Supplementary file 3 [file Table3.DOCX]
